# Understanding contrast perception in amblyopia: a psychophysical analysis of the ON and OFF visual pathways

**DOI:** 10.3389/fpsyg.2024.1494964

**Published:** 2024-10-21

**Authors:** Junhan Wei, Ziyun Cheng, Deying Kong, Wenman Lin, Robert F. Hess, Jiawei Zhou, Alexandre Reynaud

**Affiliations:** ^1^National Engineering Research Center of Ophthalmology and Optometry, Eye Hospital, Wenzhou Medical University, Wenzhou, China; ^2^State Key Laboratory of Ophthalmology, Optometry and Visual Science, Eye Hospital, Wenzhou Medical University, Wenzhou, China; ^3^Shaanxi Eye Hospital, Xi'an People’s Hospital (Xi'an Fourth Hospital), Affiliated People's Hospital of Northwest University, Xi'an, China; ^4^Department of Medical Information Management, First Affiliated Hospital of Xi'an Jiaotong University, Xi'an, Shaanxi, China; ^5^McGill Vision Research, Department of Ophthalmology and Visual Sciences, McGill University, Montreal, QC, Canada

**Keywords:** amblyopia, contrast sensitive function (CSF), qCSF, ON and OFF channels, contrast perception

## Abstract

**Purpose:**

The study aimed to explore potential discrepancies in contrast sensitivity in the ON and OFF visual pathways among individuals with amblyopia compared to controls.

**Methods:**

Eleven adult amblyopes (26.2 ± 4.4 [SD] years old) and 10 controls (24.6 ± 0.8 years old) with normal or corrected to normal visual acuity (logMAR VA ≤ 0) participated in this study. Using the quick contrast sensitivity function (qCSF) algorithm, we measured balanced CSF which would stimulate the ON and OFF pathways unselectively, and CSFs for increments and decrements that would selectively stimulate the ON and OFF visual pathways. Contrast sensitivity and area under log contrast sensitivity function were extracted for statistical analysis.

**Results:**

For the balanced CSF, we found significant interocular differences in sensitivity and area under log contrast sensitivity function in both amblyopes [*F*(1,10) = 74.992, *p* < 0.001] and controls [*F*(1,9) = 35.6, *p* < 0.001], while such differences were more pronounced in amblyopes than in controls. For increment and decrement CSFs, we found that the increment sensitivity (*p* = 0.038) and area under log contrast sensitivity function (*p* = 0.001) were significantly lower than the decrement in the amblyopic eye. Such differences between increment and decrement CSFs were not observed in the fellow eye of the amblyopes or in the controls.

**Conclusion:**

There is a subtle difference in the contrast sensitivity of the amblyopic eye when exposed to stimulation in the ON and OFF pathways.

## Introduction

Amblyopia, caused by abnormal visual experience (such as strabismus, anisometropia, or form-deprivation) during visual development (DeSantis, 2014) affects approximately 1–5% of the general population worldwide (Webber and Wood, 2005; Xiao et al., 2015). It has been shown that amblyopes not only have reduced visual acuity but also have general deficits in several visual processes, such as contrast sensitivity (Hess and Howell, 1977; Bradley and Freeman, 1981; Sjöstrand, 1981; Pang et al., 2019), binocular combination (Ding et al., 2013; Zhou et al., 2016; Mao et al., 2020), stereo vision (Levi et al., 2015), contour integration (Hamm et al., 2014), color perception (Davis et al., 2008), and so on. Consequently, amblyopia is now recognized as a developmental visual disorder of the central nervous system.

Most of the aforementioned studies measured the visual deficits of amblyopia with stimuli modulated uniformly around a mean value, typically featuring both light and dark bars in a sinusoidal profile (Ding et al., 2013; Ding and Levi, 2014; Zhou et al., 2016; Mao et al., 2020). This approach assumes that stimuli modulated in this fashion are detected by the overall amplitude of the stimulus and from what we know of visual processing in both retina and cortex, this would involve the equal contribution of both ON and OFF visual channels (Hubel and Wiesel, 1961; Schiller, 1982; Schiller et al., 1986; Schiller, 1992; Dolan and Schiller, 1994). Recently, Pons et al. (2019) studied the ON–OFF pathways sensitivity differences in amblyopia using an acuity-based paradigm. They found that there was a significant dark–light difference in accuracy and reaction time in the amblyopic eye of amblyopes. Any dark–light difference was very subtle in the fellow eye of amblyopes and normal controls. Their results indicate that amblyopia affects the ON pathway more than the OFF at suprathreshold contrast. Their measurements involve stimuli with high contrast and a discrimination task that would be expected to target extra striate function (Ziemba et al., 2019). As visual processing in the brain is very hierarchical (Grill-Spector and Malach, 2004), our question is whether the difference they observe between ON and OFF pathways could originate earlier in the visual processing stream. Indeed, studies have shown an overrepresentation of the OFF visual responses in primary visual cortex (Jin et al., 2008; Yeh et al., 2009; Xing et al., 2010). OFF processing is faster (Komban et al., 2014); and, in particular, cortical neurons are more strongly driven by darks at low spatial frequencies (Kremkow et al., 2014). Psychophysically, the most comprehensive way to understand low-level visual function is by measuring the contrast sensitivity function (Jindra and Zemon, 1989). By measuring contrast sensitivity across various spatial frequencies, we could get insight into which early processing channels could be involved in this difference. Or if we do not observe any difference, conclude a higher-order deficit. Hence, we wanted to answer two questions: (i) could the ON-selective pattern that they observed also exist at the early pathway in processing contrast sensitivity, and (ii) if so, are ON-selective deficits broad-based or spatially tuned? In current practice, the assessment of amblyopia relies mainly on high-contrast OFF stimuli, i.e., visual acuity. However, if we find ON pathway impairments at low-level visual processing stages, this may offer a novel avenue for amblyopia treatment and evaluation. For instance, selectively enhancing the ON pathway at low-levels to improve the sensitivity.

To answer these questions, we measured the contrast sensitivity of the ON and OFF visual pathways in amblyopic and control adults. Contrast sensitivity function (CSF), which describes how contrast sensitivity changes as a function of the stimulus spatial frequency (SF), determines the quality of the visual input to higher visual areas for the processing of more global and shape properties. It has been the gold standard in amblyopia research for determining whether higher-order deficits are simply the consequence of lower-order limitations (e.g., the use of a constant suprathreshold contrast). Contrast sensitivity is not only a more sensitive measure than acuity (Xiong et al., 2020) but also it provides essential information about spatial processing for lower spatial scales. In this study, we used unipolar stimuli of positive and negative polarity to explore the difference in contrast sensitivity of the ON and OFF pathways. We used the method of the quick Contrast Sensitivity Function (qCSF) (Hou et al., 2010; Lesmes et al., 2010) to measure the contrast sensitivity over different spatial frequencies.

## Materials and methods

### Participants

Eleven anisometropic amblyopes (mean age: 26.2 ± 4.4 years old; mean ± standard deviation [SD]; eight males) and 10 controls (mean age: 24.6 ± 0.8 years old; six males, including one author) with normal or corrected to normal visual acuity (0 logMAR or better) participated in this study. None of the amblyopes had any detectable ocular diseases or structural anomalies, clinical details are provided in Table 1. Amblyopia in this study was defined according to the PPP [American Academy of Ophthalmology, Preferred Practice Patterns] (Wallace et al., 2018), i.e., interocular acuity difference of more than 2 lines (0.2 logMAR) with an obvious cause like anisometropia, strabismus, or deprivation. The dominant eyes of controls were defined by the card-in-the-hole test (Dane and Dane, 2004). All subjects except one (being an author) were naive to the purpose of the experiment. Written informed consent was obtained from all participants after an explanation of the nature and possible consequences of the study. Our study has been approved by the Ethical Committee of the affiliated eye hospital of Wenzhou Medical University (ethics approval number: 2019-095-K-89) and conformed to the Declaration of Helsinki.

**Table 1:** Clinical details of amblyopes.

Subject ID	Age/sex	Cycloplegic refractive error (OD/OS)	logMAR visual acuity (OD/OS)	Squint (OD/OS)	Type	History
A1	32/F	+0.25/−0.5×130	0	Ø	OD	Detected at 11 years old, glasses and patched for 3 months since 11 years old
		+5.0/−1.0×15	0.7	Ø	OS Anis	
A2	25/M	−0.5	−0.1	Ø	OD	Detected at 18 years old, glasses and patched for 2 months since 18 years old
		+5.0/−3.0×180	0.14	Ø	OS Anis	
A3	35/M	+6.5/−1.0×10	0.84	Ø	OD Anis	Detected at 14 years old, glasses since 14 years old, no patching
		−2.5/−0.5×90	−0.08	Ø	OS	
A4	24/F	Plano	0	Ø	OD	Detected at 12 years old, no treatment
		+4.5/−0.75×8	0.5	Ø	OS Anis	
A5	21/M	−4.5	−0.1	Ø	OD	Detected at 10 years old, patched for 6 months since 11 years old
		+5.5	0.6	Ø	OS Anis	
A6	21/M	−4.0	−0.1	Ø	OD	Detected at 8 years old, patched for 6 months since 8 years old
		+5.0	0.6	Ø	OS Anis	
A7	26/F	Plano	−0.14	Ø	OD	Detected at 20 years old, no treatment
		+1.5/−0.5×180	0.22	Ø	OS Anis	
A8	30/M	−13.25/−2.0×40	0.18	Ø	OD Anis	Detected at 16 years old, glasses since 16 years old, no patching
		−1.5/−1.75×7	−0.02	Ø	OS	
A9	27/M	−4.0/−1.25×18	−0.06	Ø	OD	Detected at 14 years old, patched for 2 months since 14 years old, glasses since 14 years old
		+0.25	0.32	Ø	OS Anis	
A10	21/M	+6.25/−4.25×40	0.74	Ø	OD Anis	Detected at 6 years old, patched for 6 months
		−1.25	−0.08	Ø	OS	
A11	26/M	−16.0/−1.0×180	0.42	Ø	OD Anis	Detected at 13 years old, glasses since 16 years old, no patching
		−7.5/−0.75×10	−0.1	Ø	OS	

OD, right eye; OS, left eye; plano, emmetropia; anis, anisometropia.

### Apparatus

A PC running Matlab R2016a (Mathworks, Natick, MA, United States) with Psychtoolbox 3.0.14 (Brainard, 1997; Pelli, 1997; Kleiner et al., 2007) generated and controlled the stimuli. The stimuli were presented on a gamma-corrected cathode-ray-tube (CRT) monitor (CPD-G520, 2001, SONY Ichinomiya Corp., Japan), with a display area of 40.5 × 30.7 cm, 1,280 × 1024 pixels resolution, a refresh rate of 85 Hz, and mean background luminance of 54.5 cd/m^2^. Bits# Stimulus Processor (Cambridge Research Systems Ltd., United Kingdom) was used to generate 14-bit contrast resolution. All tests were carried out in a dark room. Before starting the test, all participants underwent dark adaptation for 5 min in the darkroom. Participants viewed the screen monocularly with a black fabric patch occluding the untested eye. A chin rest was used to minimize participants’ head movement and to ensure the viewing distance.

### Design

We measured the participant’s monocular contrast sensitivity function (CSF) using the quick contrast sensitivity function (qCSF) algorithm (Hou et al., 2010; Lesmes et al., 2010). It is a Bayesian adaptive procedure that estimates the contrast sensitivity function with a truncated log-parabola model, which is described by four parameters: gain, peak SF, bandwidth, and truncation at low spatial frequencies. This methodology has demonstrated clinical accuracy and efficiency in measuring contrast sensitivity within control and amblyopic populations (Hou et al., 2010; Gao et al., 2015). For detailed equations and descriptions, please refer to Equation 1 in the Supplementary material.

For each participant, the monocular CSFs were measured with three types of stimuli: balanced contrast, increment, and decrement patterns. These three types of stimuli were tested in a randomized order to eliminate the possible learning effect. The 6 CSFs (3 stimuli types × 2 eyes) were tested twice in the same day, and the results were averaged based on the two repetitions. Each CSF was measured in 100 trials, preceded by 5 practice trials with full-contrast (100%) stimuli. It took about 5 min for each participant to complete one CSF measure. After finishing two CSF measures (i.e., ~10 min) with one eye, the participant was asked to take a 2-min rest before proceeding to test the other eye with two additional CSF measures.

### Stimuli

**Figure 1:**
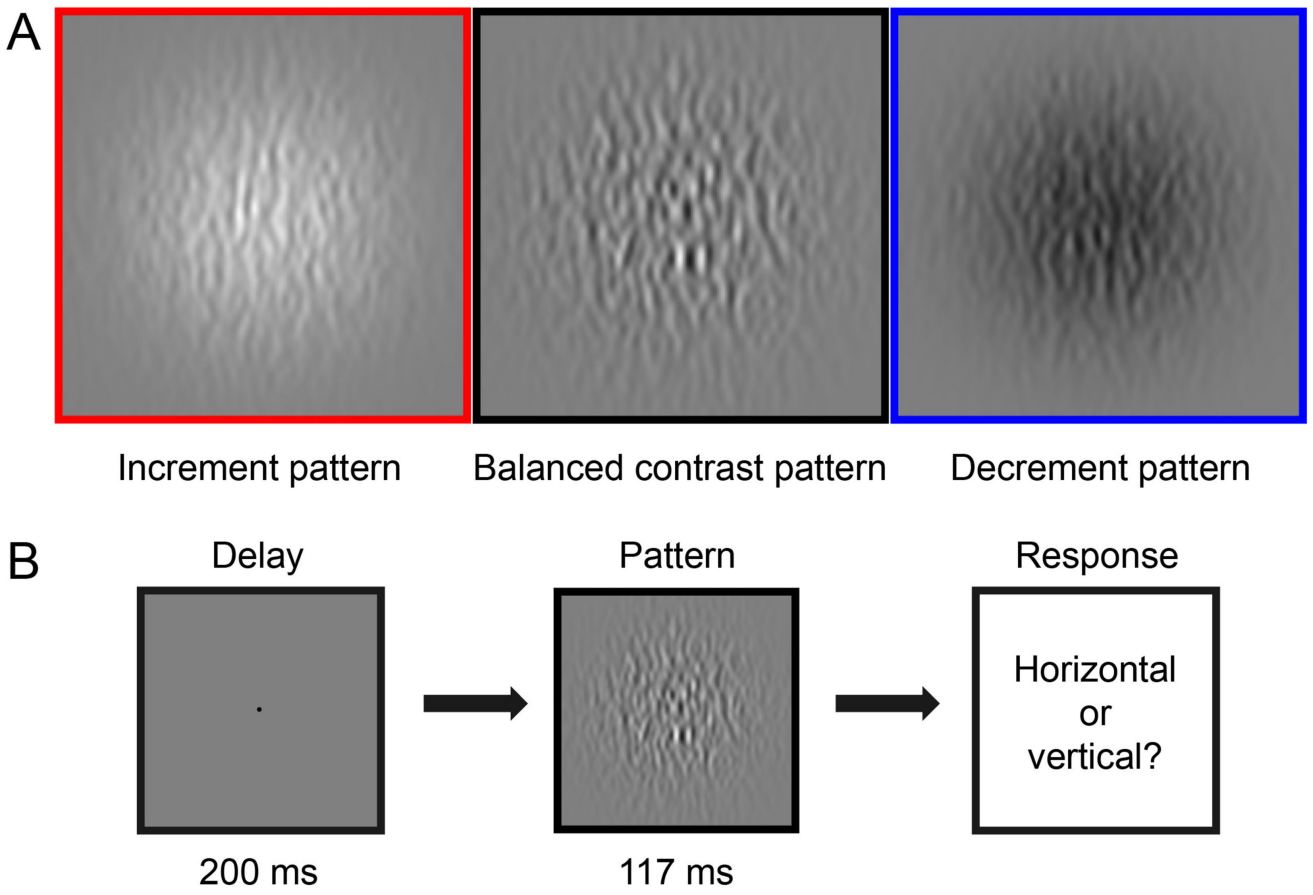
Illustration of the experiment design. **(A)** Three types of visual stimuli in this study: increment pattern (red box, positive-contrast stimuli), balanced contrast pattern (black box), and decrement pattern (blue box, negative-contrast stimuli). **(B)** Each trial started with a 200-ms presentation of the black fixation point in the center of the gray background, followed by a 117-ms presentation of the test stimuli. Observers were asked to report the orientation of the stimulus in each trial, i.e., horizontal or vertical.

Figure 1A illustrates the three types of stimuli we generated for the CSF tests. The stimuli consisted of horizontal or vertical bandpass filtered noise in a Gaussian window. They were generated by filtering white noise in the spatial domain with a Gabor filter at the desired spatial frequency (bandwidth of 1.84 octaves). The three stimuli types included: (1) Balanced contrast pattern (as shown in the black box in Figure 1A) aimed to simultaneously stimulate both ON and OFF pathways in the CSF measurement (Kim et al., 2017). (2) Increment pattern (illustrated in the red box in Figure 1A) presented positive contrast (brighter filtered noise than the background) to selectively stimulate the ON pathway. (3) Decrement pattern (displayed in the blue box in Figure 1A) provided negative contrast (darker filtered noise than the background) to selectively stimulate the OFF pathway. Amblyopic individuals viewed stimuli in a Gaussian window with a sigma of 10°, while control subjects viewed stimuli in a Gaussian window with a sigma of 4°. Amblyopes have impaired contrast sensitivity in high SF compared to normal individuals (Hess and Howell, 1977; Sjöstrand, 1981). This implies that these two groups would present different cutoff spatial frequencies (Volkers et al., 1987; Majander et al., 2017). Based on these previous reports, we optimized the testing range to [0.31–32.31] c/d for the control group and [0.31–11.77] c/d for the amblyopic group. Spatial frequency (SF) and contrast levels were varied by the qCSF approach (Lesmes et al., 2010) in different trials. Moreover, to maintain comparable computational times for processing stimuli (in terms of pixels), different viewing distances (57 cm for amblyopes and 140 cm for normal controls) were employed. This variation in viewing distance had minimal impact on their light adaptation levels, given the negligible pupillary changes within this range (Lin et al., 2022).

### Procedure

As shown in Figure 1B, in one CSF trial, a black fixation point (radius 0.15°), signaled by a brief tone to begin, was presented in the center of the screen for 200 milliseconds (ms). This was followed by a 117 ms presentation of a horizontal or vertical (randomized in each trial) test stimulus. Participants were asked to identify whether the orientation of the stimulus was horizontal or vertical by pressing the ‘left’ or ‘up’ key on the keyboard. The next trial started immediately after the response.

### Statistical analysis

We extracted various parameters for statistical analysis, including contrast sensitivities at chosen spatial frequencies (SF), the area under log contrast sensitivity function (AULCSF; the integration of the log-parabola over the entire SF range of the measurements), cut-off SF corresponding to a contrast threshold of 0.5, and the four qCSF parameters (gain, peak SF, bandwidth, and truncation at low SF). Repeated-measures analysis of variances (ANOVAs), Bonferroni-corrected Pairwise Comparisons, *t*-tests, and Pearson correlation tests were used to compare the difference and correlation of contrast sensitivity, AULCSF, cut-off SF, qCSF parameters and the interocular difference between the three testing conditions (i.e., balanced contrast, decrement and increment patterns). Wilcoxon signed-rank tests were used to compare the differences between the ratio of increment/decrement contrast sensitivity and 1. The analysis of the cut-off SF and qCSF parameters are presented in Supplementary Figures S1, S2. Statistical analyses were performed using IBM-SPSS 23.0 (IBM Inc., Armonk, NY, United States).

## Results

### The balanced contrast sensitivity

**Figure 2:**
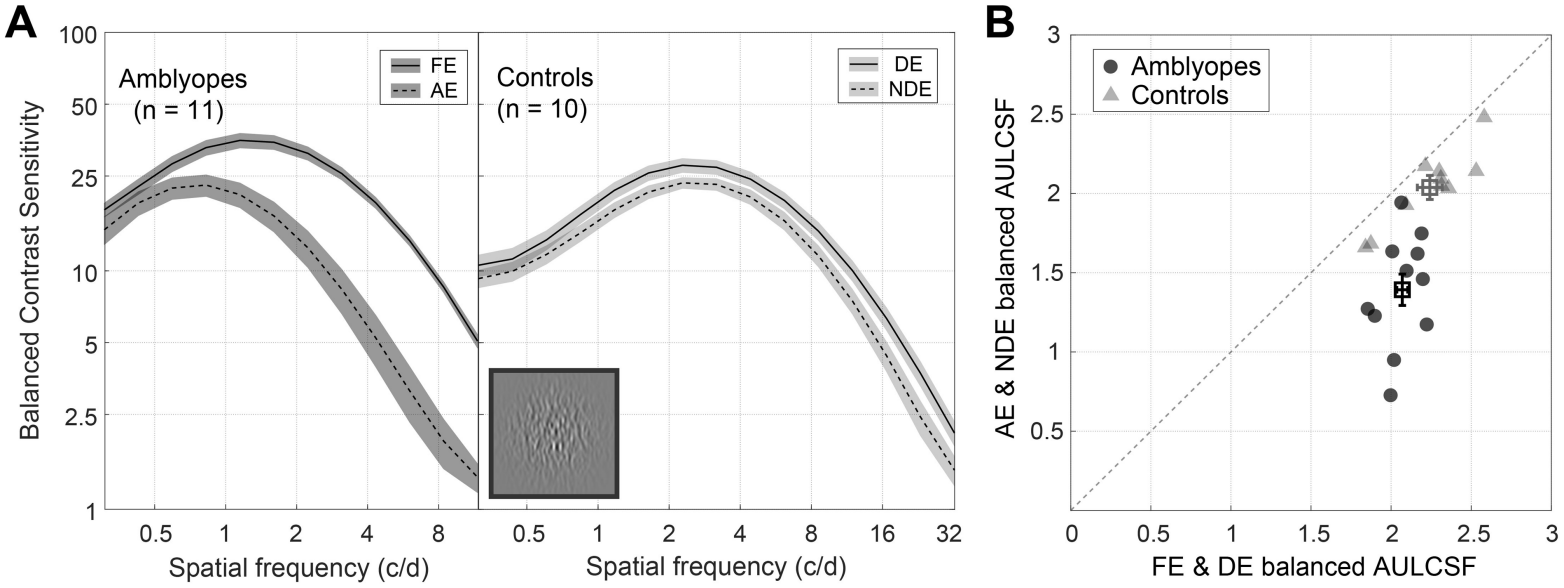
Balanced contrast sensitivity of amblyopes and controls. **(A)** Average balanced CSF of amblyopes (left) and controls (right). The solid lines represent the fellow eye (FE) and dominant eye (DE), and the dashed lines represent the amblyopic eye (AE) and non-dominant eye (NDE). The shaded regions represent ± standard error [SE]. **(B)** Average balanced the area under the log contrast sensitivity function (AULCSF) of amblyopes (circles) and controls (triangles), *X*-axis represents FE and DE, and *Y*-axis represents AE and NDE. Each symbol represents one subject. The average results were plotted with square symbols. The dashed line represents the identity. Error bars represent SE. A direct comparison between amblyopes and controls is not possible due to the difference in experimental design.

Figure 2A shows the average CSFs for 11 amblyopes (left) and 10 controls (right) under the balanced contrast test condition. As expected, the amblyopic group exhibited a large extent of interocular difference in balanced contrast sensitivity across spatial frequencies, whereas the control group displayed minimal interocular differences. A two-way repeated-measures analysis of variance (ANOVA) revealed that the contrast sensitivity was significantly different between eyes in both amblyopes [*F*(1,10) = 74.992, *p* < 0.001, partial ${ŋ}^{2}$ = 0.882] and controls [*F*(1,9) = 35.561, *p* < 0.001, partial ${ŋ}^{2}$ = 0.789]. In Figure 2B, we plotted individuals’ area under the log contrast sensitivity function (AULCSF) for the amblyopic eye (or non-dominant eye) as a function of the fellow eye (or dominant eye). Notably, the datapoints of controls (triangles) cluster around the identity line, the average ratio of the area under the log contrast sensitivity function for the non-dominant eye to the dominant eye was 0.91. In contrast, the majority of amblyopic individuals’ data points (circles) fall below the identity line, the average ratio of the area under the log contrast sensitivity function for the amblyopic eye to the fellow eye was 0.67. Paired-samples t-tests confirmed the statistical significance of interocular differences in balanced area under the log contrast sensitivity function for both amblyopes (*t* = 6.677, *p* < 0.001, Cohen’s *d* = 2.986) and controls (*t* = 6.272, *p* < 0.001, Cohen’s *d* = 2.957). Additionally, in Supplementary Figure S4, we provide evidence of a linear predictive relationship between the balanced CSF and the decrement and increment CSF. We did not directly compare results in the amblyopic and control groups because of the different stimulus sizes and testing distances employed.

### The difference between decrement and increment contrast sensitivity

**Figure 3:**
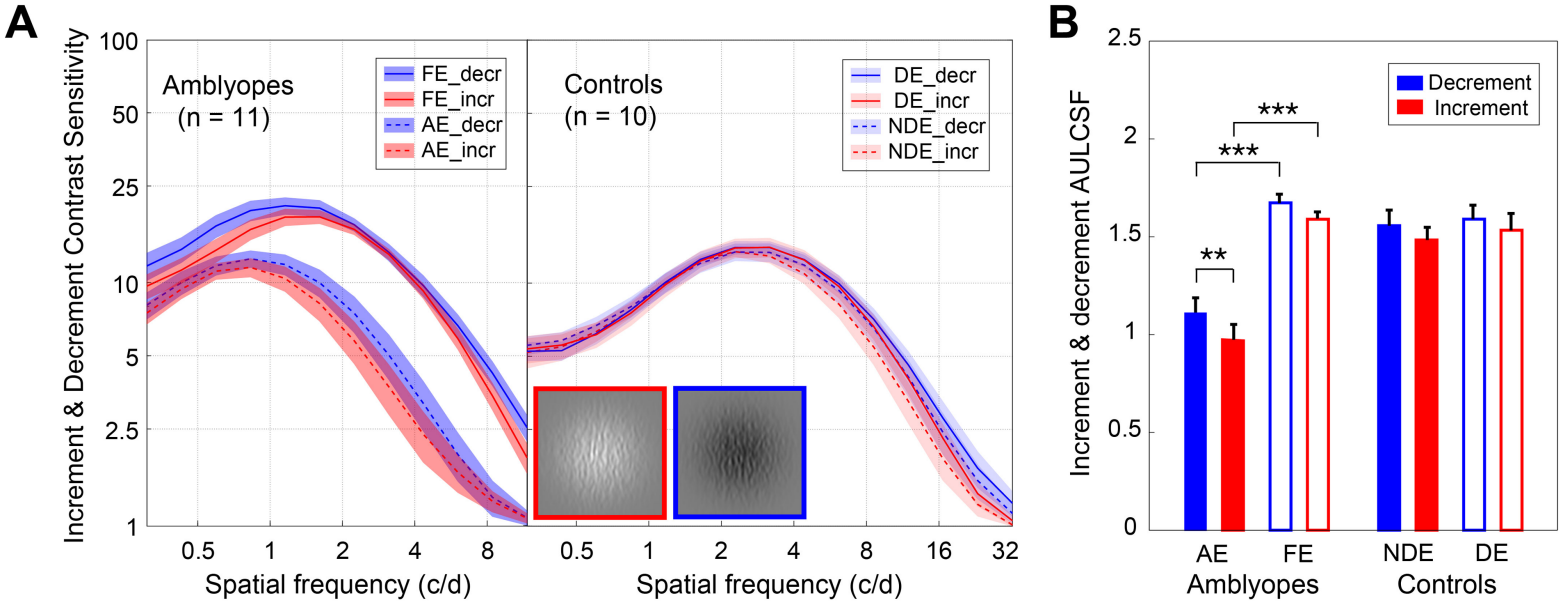
Decrement and increment contrast sensitivities of amblyopes and controls. **(A)** Average decrement (blue) and increment (red) CSF of amblyopes (left) and controls (right). The solid lines represent the FE (fellow eye) and NDE (non-dominant eye), the dashed lines represent the AE (amblyopic eye) and DE (dominant eye). The shaded areas represent ± SE. **(B)** The average area under the log contrast sensitivity function for decrement (blue) and increment (red) of amblyopes (left) and controls (right). The solid bars represent AE and NDE, the hollow bars represent FE and DE. Error bars represent SE. Results of the repeated measures ANOVA are shown: *p < 0.05, **p < 0.01, ***p < 0.001. A direct comparison between amblyopes and controls is not possible due to the difference in experimental design.

In Figure 3A, we have depicted the average decrement and increment CSFs for both amblyopes and controls. As anticipated, both decrement and increment CSFs were significantly lower than the balanced CSF (*p* < 0.001, for all). For amblyopes (Figure 3A, left), contrast sensitivities of the amblyopic eye were lower in both the decrement (blue curves) and increment (red curves) conditions compared to the fellow eye. However, no such interocular difference was found in controls (Figure 3A, right). Indeed, the area under the log contrast sensitivity function for the AE were significantly lower in both the two conditions for amblyopes (*p* < 0.001, for all; Figure 3B), while no interocular area under the log contrast sensitivity function differences were observed in controls.

Interestingly, we found that the increment CSF was slightly lower than that for the decrement in the amblyopic eye of amblyopes (Figure 3A). Two three-way repeated-measures ANOVAs, with test condition (2 levels: decrement and increment), eye (2 levels), and spatial frequency (12 levels) as within-subject factors were conducted for the two groups, respectively. In amblyopes, we identified significant main effects for eye [*F*(1,10) = 56.059, *p* < 0.001, partial ${ŋ}^{2}$ = 0.849], condition [*F*(1,10) = 4.244, *p* = 0.066, partial ${ŋ}^{2}$ = 0.298], and spatial frequency [*F*(1.7,17.1) = 71.864, *p* < 0.001, partial ${ŋ}^{2}$ = 0.878]. We also observed a significant interaction between eye and spatial frequency [*F*(1.7,16.7) = 13.383, *p* = 0.001, partial ${ŋ}^{2}$ = 0.572]. However, we found no significant interactions between eye and condition [*F*(1,10) = 0.715, *p* = 0.418, partial ${ŋ}^{2}$ = 0.067], condition and spatial frequency [*F*(1.6,16.0) = 2.484, *p* = 0.123, partial ${ŋ}^{2}$ = 0.199], or the three-way interaction between eye, condition and spatial frequency [*F*(1.8,18.0) = 1.697, *p* = 0.212, partial ${ŋ}^{2}$ = 0.145]. Subsequent Bonferroni-corrected pairwise comparisons revealed a significant difference in contrast sensitivity between decrement and increment in the amblyopic eye (*p* = 0.038), while such a difference was not significant in the fellow eye (*p* = 0.133). Conversely, in the control group, we found a significant main effect for spatial frequency [*F*(1.7,19.0) = 114.359, *p* < 0.001, partial ${ŋ}^{2}$ = 0.927], but not for eye [*F*(1,9) = 1.513, *p* = 0.25, partial ${ŋ}^{2}$ = 0.144] or condition [*F*(1,9) = 3.702, *p* = 0.087, partial ${ŋ}^{2}$ = 0.291]. Furthermore, we found no significant interaction between eye and condition [*F*(1,11) = 0.116, *p* = 0.741, partial ${ŋ}^{2}$ = 0.013], eye and spatial frequency [*F*(1.7,18.5) = 1.599, *p* = 0.088, partial ${ŋ}^{2}$ = 0.151], or eye and condition and spatial frequency [*F*(1.8,20.0) = 0.318, *p* = 0.991, partial ${ŋ}^{2}$ = 0.034]. Further Bonferroni-corrected pairwise comparisons indicated little difference in contrast sensitivity between decrement and increment in non-dominant eye (*p* = 0.272) or dominant eye (*p* = 0.668).

We further compared the area under the log contrast sensitivity function between the two test conditions (Figure 3B) by conducting two-way repeated-measures ANOVAs, with test condition (2 levels: decrement and increment) and eye (2 levels) as the within-subject factors for the two groups. In amblyopes, we observed significant main effects for eye [*F*(1,10) = 59.603, *p* < 0.001, partial ${ŋ}^{2}$ = 0.856] and test condition [*F*(1,10) = 14.828, *p* = 0.003, partial ${ŋ}^{2}$ = 0.597]. Bonferroni-corrected pairwise comparisons disclosed a significant difference between decrement and increment conditions in amblyopic eye (*p* = 0.001), but not in fellow eye (*p* = 0.107), with the disparity characterized by higher cut-off spatial frequencies in the decrement CSF (Supplementary Figure S2A). Among the control group, a significant main effect was observed for the test condition [*F*(1,9) = 8.783, *p* = 0.016, partial ${ŋ}^{2}$ = 0.494], yet Bonferroni-corrected pairwise comparisons showed no significant differences between decrement and increment conditions for either non-dominant eye (*p* = 0.144) or dominant eye (*p* = 0.333).

**Figure 4:**
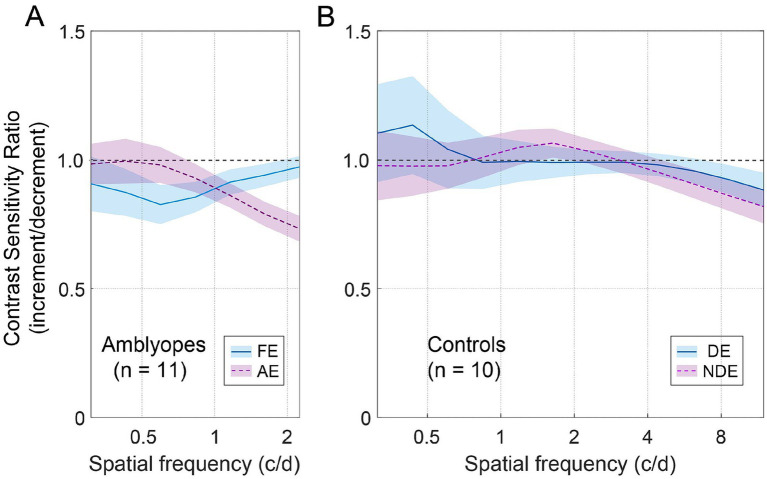
The average contrast sensitivity ratio of increment/decrement. **(A)** Average ratio for amblyopes. The blue solid line represents FE (fellow eye), and the dashed purple line represents AE (amblyopic eye). The shaded regions represent ± SE. **(B)** Average ratio for controls. The blue solid line represents DE (dominant eye), and the dashed purple line represents NDE (non-dominant eye).

To illustrate the joint effect of spatial frequency and the increment and decrement test conditions on observers’ contrast sensitivities, we computed the ratio of increment/decrement contrast sensitivity, and presented this ratio as a function of spatial frequency in Figure 4. To ensure accuracy and mitigate any potential floor effects due to stimuli visibility limitations at high spatial frequencies, we focused solely on the spatial frequency range where corresponding contrast thresholds were less than 1, namely 0.31–2.25 c/d for amblyopes, and 0.31–11.96 c/d for controls. As depicted in Figure 4A, the ratio of increment/decrement contrast sensitivity fell below the unity line and decreased with increasing spatial frequency in the amblyopic eye (purple dashed line). Such pattern was not observed in the fellow eye of amblyopes (Figure 4A, blue solid line) or in the eyes of controls (Figure 4B), where the ratios remained close to 1 across all spatial frequencies. We then conducted two-way ANOVAs with eye and spatial frequency as within-subject factors for the two groups, respectively. These analyses did not reveal any significant main effects for eye or spatial frequency in either group (*p* > 0.5, for all). However, among amblyopic individuals, a noteworthy interaction between eye and spatial frequency emerged regarding the ratio of increment/decrement contrast sensitivity [*F*(1.8,17.5) = 3.735, *p* = 0.049, partial ${ŋ}^{2}$ = 0.272]. In contrast, controls did not exhibit a significant interaction between eye and spatial frequency [*F*(2.0,18.4) = 0.459, *p* = 0.643, partial ${ŋ}^{2}$ = 0.049].

Furthermore, we found that in the amblyopic eye, the ratio of increment/decrement contrast sensitivity was significantly smaller than 1 at spatial frequencies of 1.16 c/d (Wilcoxon signed-rank test: *Z* = 2.134, *p* = 0.033), 1.61 c/d (*Z* = 2.845, *p* = 0.004), and 2.24 c/d (*Z* = 2.934, *p* = 0.003). And in the fellow eye, the ratio was significantly smaller than 1 at a spatial frequency of 0.83 c/d (*Z* = 2.04, *p* = 0.041). For controls, the ratio was significantly smaller than 1 at spatial frequencies of 8.58 c/d (*Z* = 2.293, *p* = 0.022) and 11.96 c/d (*Z* = 2.395, *p* = 0.017).

### The relationship between the decrement and increment contrast sensitivities

**Figure 5:**
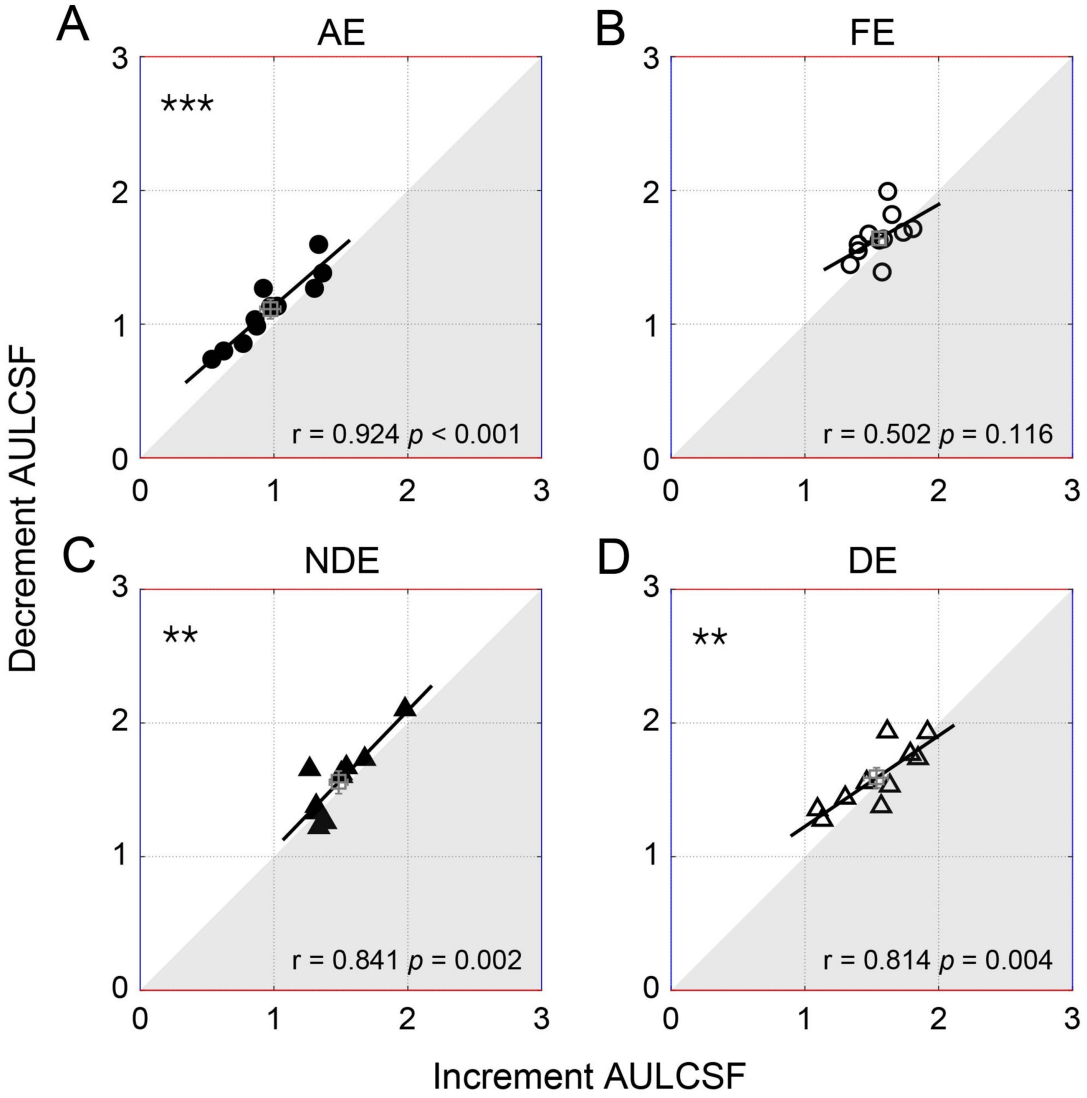
The individual decrement area under the log contrast sensitivity function (AULCSF) as a function of increment for amblyopes (circles, the top row) and controls (triangles, the bottom row). Each symbol represents one subject. The average results were plotted with the square symbol; error bars represent SE. The solid lines represent the best linear fits. Results of Pearson correlation test are shown: #*p* < 0.1, **p < 0.01, ***p < 0.001. **(A)** AE: amblyopic eye, **(B)** FE: fellow eye, **(C)** NDE: NON-dominant eye, **(D)** DE: dominant eye.

To further illustrate the relationship between the decrement and increment contrast sensitivities, we plotted the individual area under the log contrast sensitivity function of the decrement condition as a function of the increment condition in Figure 5. Pearson correlation analysis indicated a significant positive correlation between the area under the log contrast sensitivity function of the two conditions. This correlation was observed to be significant in the amblyopic eye (*r* = 0.924, *p* < 0.001) and in both two eyes of controls (*p* < 0.005, for all), although it was not significant in the fellow eye (*p* = 0.116). A similar relationship was observed among all qCSF parameters (Supplementary Figure S1) and the cut-off spatial frequency (Supplementary Figure S2B).

## Discussion

In this study, we explored the contrast sensitivity of ON and OFF visual pathways in adult amblyopic patients and control adults, using positive-contrast stimuli that activate the ON visual pathway (increment pattern), negative-contrast stimuli activating the OFF visual pathway (decrement pattern), and stimuli activating both pathways simultaneously (balanced contrast pattern). Our findings revealed that contrast sensitivities for both increment and decrement conditions were lower than those for balanced contrast sensitivity across all eyes. Interestingly, in the amblyopic eye, the sensitivity to increment stimuli was lower than that for decrement sensitivity at high spatial frequencies. Furthermore, we observed a positive correlation between sensitivities in these two conditions. These results suggest that while amblyopes exhibit reduced sensitivity in both ON and OFF pathways, the contrast sensitivity loss in the ON visual pathway is marginally greater than that in the OFF pathway at higher spatial frequencies.

In mammals, the ON and OFF visual pathways originate from the bipolar cells of the retina (Masu et al., 1995; Vardi and Morigiwa, 1997). OFF cells, characterized by their higher number, smaller dendritic fields, and denser branching, likely reflect the more negative contrast information present in natural scenes (van Hateren et al., 2002; Ratliff et al., 2010). These two pathways transmit brightness and darkness information separately to the LGN (Hubel and Wiesel, 1961; Kaplan and Shapley, 1982), and subsequently to the primary visual cortex (McConnell and LeVay, 1984; Zahs and Stryker, 1988; Kremkow et al., 2016), where they converge on individual V1 neurons to form receptive fields (Alonso et al., 2001). The dominance of OFF cells in cortical responses is attributed to their wider thalamic input converge, stronger connections, and greater excitatory afferents compared to ON cells (Jin et al., 2008; Yeh et al., 2009; Jin et al., 2011). In addition, studies such as Luo-Li et al. (2016) have highlighted the ON–OFF difference in detecting light and dark bars, with individuals responding more accurately and faster to dark stimuli, consistent with other psychophysics studies (Bowen et al., 1989; Chubb and Nam, 2000; Buchner and Baumgartner, 2007; Komban et al., 2011; Komban et al., 2014).

Controversy exists in studies on ON–OFF perception in normal individuals. To illustrate, Barboni et al. (2013) showed that ON contrast sensitivity was lower than OFF contrast sensitivity, using sawtooth stimuli at 0.3 and 2 c/d. While, Komban et al. (2011) measured the contrast thresholds of normal subjects at 16, 24, and 32 c/d using square-wave grating, and found that the contrast perception of the ON pathway was slightly reduced compared with that of the OFF, but this difference was not significant. Our findings align more with the latter, indicating slightly larger area under the log contrast sensitivity function in the decrement condition in the non-dominant eye compared to the increment condition without statistical significance. Future research might explore the spatial frequency dependency of this comparison further.

In adults with amblyopia, we noted a slightly larger deficit in contrast sensitivity for the ON pathway stimulation, positively correlated with the OFF-pathway deficit. Amblyopes generally exhibit lower contrast sensitivity compared to controls, particularly at high spatial frequencies (Hess and Howell, 1977; Sjöstrand, 1981). This means that the stimulus contrast at threshold (and particularly at high spatial frequencies) is higher when testing the amblyopic eye compared to the fellow eye or the normal controls. While at high contrast, there is more perceptual difference between light and dark targets (Wallis et al., 2013). This is consistent with previous studies, notably Pons et al.’s study (2019), which indicated a larger deficit for ON stimulation compared to OFF at suprathreshold contrast levels. They revealed that for this high-level perceptual task, amblyopia exhibits a larger deficit for ON compared with OFF stimulation. Our study’s results at the detection threshold are in harmony with these findings, showing a slightly greater deficit for ON stimulation than OFF in the amblyopic eye and a positive correlation between the two pathways.

As for the interocular difference (i.e., the difference between amblyopic eye and fellow eye, and the difference between non-dominant eye and dominant eye), we found that the interocular differences of area under the log contrast sensitivity function in amblyopes were larger than those of controls in both ON and OFF pathways (Supplementary Figure S3B). Notably, a smaller difference in the ON pathway correlated with a reduced difference in the OFF pathway in amblyopic eyes, a correlation not seen in normal subjects (Supplementary Figures S3B,C). Our study indicates that the ON–OFF difference in amblyopia already exists in the early stages of contrast processing (Owsley, 2003). This impairment in low-level, high spatial frequency contrast detection may impact higher processing stages, potentially affecting the perception of lower spatial frequency suprathreshold stimuli (e.g., see Pons et al., 2019) due to the convergence of low-level small receptive fields at these stages.

There is a potential effect of the phantom array (Jordan and Hershberger, 1994; Hershberger et al., 1998) that has been reported mainly at the neuronal level but has been neglected in the psychophysical literature. However, we do not believe that this effect can explain the ON-selective deficit reported in Pons’ study (2019) and the pattern we show here. First, if this effect selectively affected the percept of ON stimuli, we would expect a similar ON-selective deficit in all participants. However, we found selective deficits in ON percept only in amblyopic eyes and not in fellow eyes or normal controls. Second, none of our participants reported such percepts which we believe was due to two things: first, we instructed all participants to fixate during stimulus presentation; second, the target stimulus was not strongly periodic with sharp edges, it was a filtered texture with no sharp edges. Another limitation to consider is the different experimental designs used for amblyopes and controls. While a larger viewing distance was used to test higher spatial frequencies in the control group, this difference did not allow us to directly compare the results between amblyopes and controls (Figures 2, 3). This viewing distance difference would not significantly impact participants’ light adaptation (Lin et al., 2022). And, as other testing conditions were similar, we believe that it should not unduly affect our measurements and conclusions. Additionally, it is worth noting that we used a small black fixation point (radius 0.15°) in our tests to guide subjects to the stimulus location. This fixation point disappeared once the stimulus appeared, ensuring it did not influence the experimental results.

## Conclusion

Current diagnosis, treatment, or clinical trials of amblyopia mainly focus on the improvement of visual acuity. Visual acuity is measured usually by asking patients to identify high-contrast black letters on a white background, which is biased toward OFF pathway stimulation. We show that amblyopia affects both the ON and OFF visual pathways at an early level of visual processing, and that amblyopes exhibit a slight difference between the ON and OFF visual pathways. This discovery represents a significant stride toward comprehending the development of ON and OFF visual pathways, potentially contributing to refining amblyopia assessment and treatment strategies.

## Data Availability

The datasets presented in this study can be found in online repositories. The names of the repository/repositories and accession number(s) can be found: Github repository (https://github.com/junhan16/amblyopia.git).
