# Supplementary material for: Understanding contrast perception in amblyopia: a psychophysical analysis of the ON and OFF visual pathways
Source: Front Psychol. 2024 Oct 21;15:1494964. doi: 10.3389/fpsyg.2024.1494964 (PMC11532024; doi:10.3389/fpsyg.2024.1494964)
Supplement: Supplementary file 1 [file Presentation_1.pdf]

# Supplementary Materials

## The difference between decrement and increment qCSF parameters

To further show the difference between how the ON and OFF visual pathways are affected by amblyopia, we analyzed the qCSF parameters in response to the increment and decrement test conditions. The qCSF method has four parameters: (1) $\gamma_{max}$, the peak gain (sensitivity); (2) $f_{max}$, the peak spatial frequency; (3) $\beta$, the bandwidth, which describes the function's full-width at half-maximum (in octaves), and (4) $\delta$, the truncation level at low spatial frequencies. The equation of the qCSF is:

$$f_1(x) = log_{10}(\gamma_{max}) - \delta$$

$$f_2(x) = log_{10}(\gamma_{max}) + log_{10}(0.5)\left(\frac{log_{10}(x) - log_{10}(f_{max})}{\beta * log_{10}(2)/2}\right)^2$$

$$f(x) = \begin{cases} f_1(x), & f_2(x) < f_1(x) \; and \; log_{10}(x) < log_{10}(f_{max}) \\ f_2(x), & f_2(x) \geq f_1(x) \; or \left( log_{10}(x) > log_{10}(f_{max})\right) \end{cases}, \quad (1)$$

where the $f(x)$ is $log_{10}(\mathrm{CSF})$, x is spatial frequency.

Supplementary Fig. S1 shows decrement qCSF parameters as a function of increment qCSF parameters for amblyopes (circles) and controls (triangles). For the four parameters, no significant differences were found in all eyes ($p$ > 0.05, for all). Spearman correlation tests for two conditions showed that in AE, all increment and decrement qCSF parameters except truncation were positively correlated ($p$ < 0.02, for all). While in other eyes, the correlation was not consistent, the positive correlation between the two conditions we found was mainly reflected in AE.

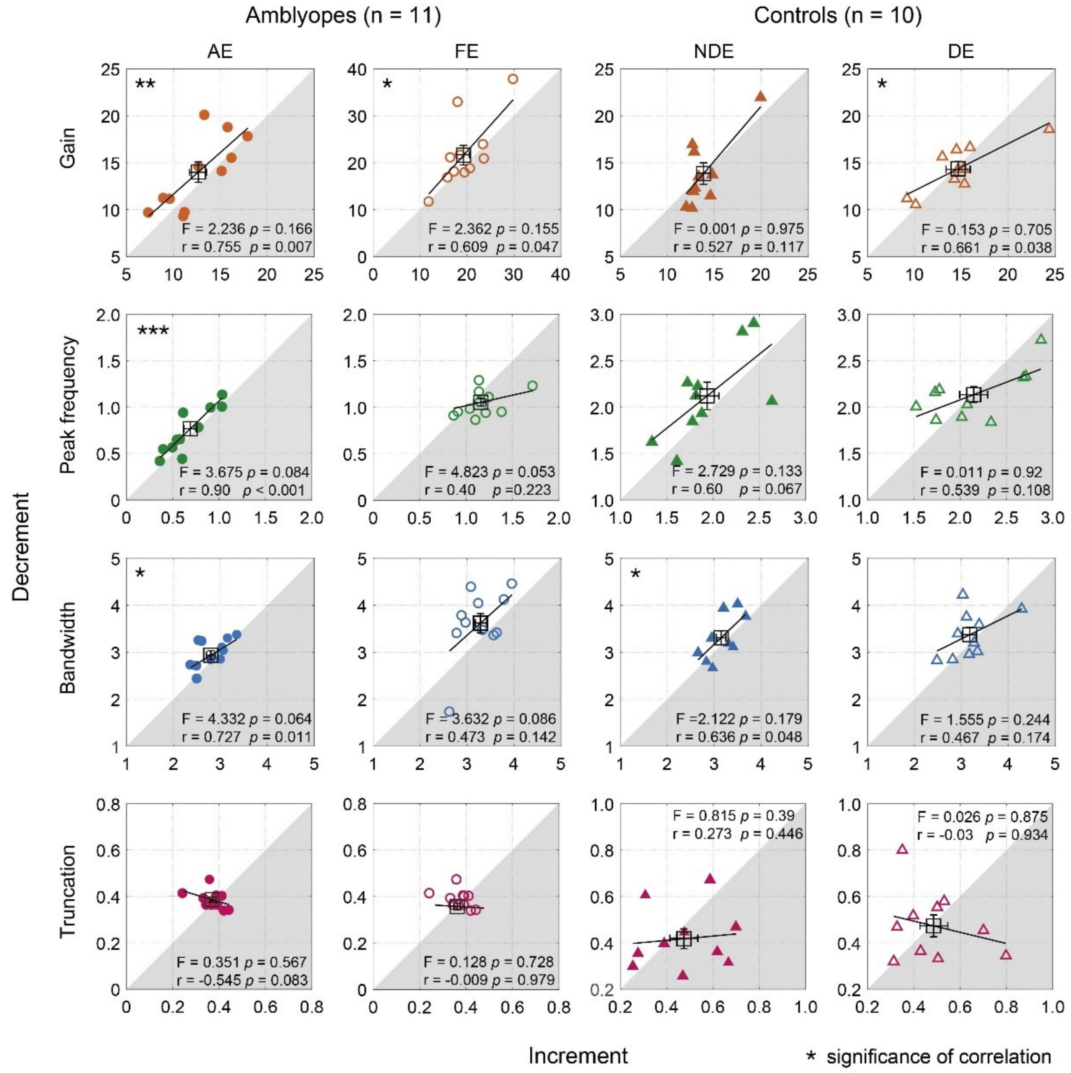


**Fig. S1.** The decrement qCSF parameters (i.e., gain, peak frequency, bandwidth, and truncation) as a function of increment qCSF parameters for amblyopes (left, circles) and controls (right, triangles). Each symbol represents one subject. Average values are represented by a square symbol; error bars represent SE. Repeated measures ANOVA and Spearman correlation results are shown. The black lines represent the best linear fittings. Significance of correlation are shown: * $p < 0.05$, ** $p < 0.01$, *** $p < 0.001$. AE, amblyopic eye; FE, fellow eye; NDE, non-dominant eye; DE, dominant eye.

## The decrement and increment cut-off SF

Similar to previous studies (Zhou et al. 2006; Huang et al. 2007; Hou et al. 2010),

the cut-off spatial frequency of the qCSF was defined as the spatial frequency at which contrast sensitivity drops to 2.0 (threshold = 0.5). As can be seen in Supplementary Fig. S2A, in all eyes of the two groups, the cut-off SF for the decrement condition was higher than those of the increment. Repeated measures ANOVA showed that such difference was significant in amblyopic eye ($F [1,10] = 11.757$, $p = 0.006$, partial $\eta^2 = 0.540$), fellow eye ($F [1,10] = 4.938$, $p = 0.051$, partial $\eta^2 = 0.331$), and non-dominant eye ($F [1,9] = 9.125$, $p = 0.014$, partial $\eta^2 = 0.503$). Supplementary Fig. S2B shows the relationship between cut-off SFs of the two conditions. Pearson correlation test showed that there was a significant positive correlation between decrement cut-off SF and increment cut-off SF in all eyes ($p < 0.025$, for all).

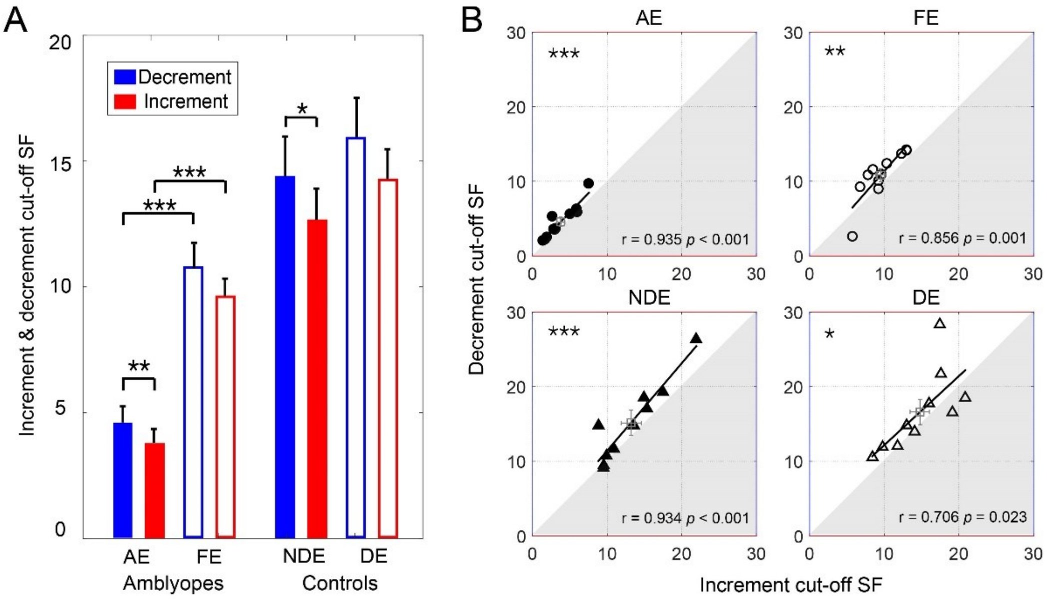


**Fig. S2. Decrement and increment cut-off SF for amblyopes and controls.**

**(A)** Average decrement (blue) and increment (red) cut-off SF for amblyopes (left) and controls (right). The solid bars represent amblyopic eye (AE) and non-dominant eye (NDE), and the hollow bars represent fellow eye (FE) and dominant eye (DE). Error bars represent SE. Results of repeated-measures ANOVA are shown: * $p < 0.05$, ** $p < 0.01$, *** $p < 0.001$. **(B)** The decrement cut-off SF as a function of increment cut-off SF for amblyopes (circles) and controls

(triangles). Each symbol represents one subject. Average values are represented by square symbols; error bars represent SE. The solid lines represent the best linear fittings. Results of Pearson correlation test are shown: ** $p < 0.01$; *** $p < 0.001$.

**Interocular difference between the decrement and increment conditions**

So far, we have analyzed the contrast sensitivity difference between the decrement and increment conditions for different eyes in the two groups. In this section, we compare the contrast sensitivity difference between the two eyes for the decrement and increment testing conditions. We analyzed the difference of the interocular ratio (AE/FE for amblyopes, NDE/DE for controls) of contrast sensitivity between increment and decrement conditions. To avoid floor effect, we only analyzed the data at the SF range of 0.31 to 2.25 c/d where corresponding contrast thresholds were less than 1 for all amblyopes, and two-way ANOVAs with condition and SF as within-subject factors were conducted. There was a significant main effect of SF ($F [1.8,20.8] = 20.756$, $p < 0.001$, partial $\eta^2 = 0.675$) for amblyopes, while no significant main effect of condition or interaction was found.

Supplementary Fig. S3A shows that the interocular ratio of amblyopes was lower than that of controls for both decrement and increment conditions (independent-sample t-test: $p < 0.001$, for all), which means that the interocular differences of amblyopes were larger than those of controls. And as illustrated in Supplementary Fig. S3B, most of the amblyopes datapoints (circles) are above the identity line, indicating that the AE/FE AULCSF of decrement in most amblyopic participants was larger than increment (paired samples t-test: $t = 1.808$, $p = 0.101$, cohen's $d = 0.324$). And Pearson correlation showed that they were positively correlated ($r = 0.869$, $p = 0.001$). Yet, for controls (Fig. S3C), the triangles were evenly distributed around 1, and no difference nor correlation was found ($p > 0.4$, for all).

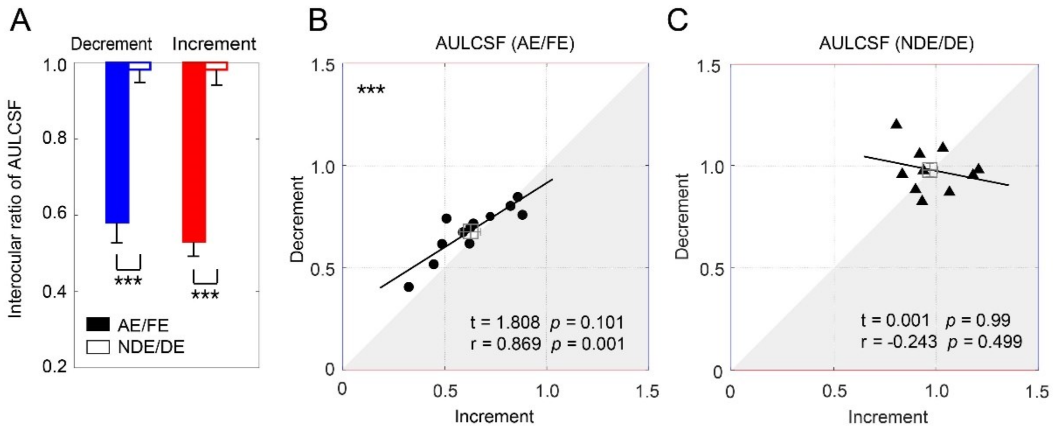


**Fig. S3. The interocular difference for amblyopes and controls.**

**(A)** The interocular ratio of AULCSF under decrement (blue) and increment (red) of amblyopes (solid bars) and controls (hollow bars). Error bars represent SE. Results of independent sample t-tests are shown: *** $p < 0.001$. **(B, C)** The interocular ratio of the decrement AULCSF as a function of the increment for amblyopes (B, AE/FE) and controls (C, NDE/DE). Each symbol represents one subject: circle, amblyope; triangle, control. Average values are indicated with a square symbol; error bars represent SE. Paired samples t-test and Pearson correlation results are shown. Solid lines represent linear regressions. Results of Pearson correlation are shown: *** $p < 0.001$.

## Balanced contrast sensitivity can be predicted linearly from decrement and increment contrast sensitivities

To test whether the balanced contrast sensitivity can be derived from the decrement and increment sensitivities, in Supplementary Fig. S4, we plotted the balanced CSF (gray) and the CSF resulting from the linear summation from decrement and increment CSF (pink) for amblyopes (left panel) and controls (right panel). To avoid floor effects, we only analyzed the data in the SF range where contrast thresholds were less than 1, that is, 0.31 to 2.25 c/d for amblyopes, and 0.31 to 11.96 c/d for controls. These two curves are almost overlapping for all eyes. Repeated measures ANOVAs with the eye,

condition (i.e., balanced contrast and decrement + increment), and SF as within-subject factors for the two groups were conducted, respectively. In amblyopes, we found significant main effects for eye ($F$ [1,10] = 34.498, $p < 0.001$, partial $\eta^2 = 0.775$), condition ($F$ [1,10] = 8.424, $p$ =0.016, partial $\eta^2 = 0.457$), and SF ($F$ [1.3,13.4] = 27.553, $p < 0.001$, partial $\eta^2 = 0.291$), whereas there was a significant interaction between eye and SF ($F$ [1.5,15.4] = 21.721, $p < 0.001$, partial $\eta^2 = 0.734$). We then performed Bonferroni-corrected pairwise comparisons, and we found there was a significant difference between balanced CSF and linear summation of the decrement and increment CSF in FE ($p = 0.034$), but not in AE ($p = 0.269$). In controls, we found a significant main effect for eye ($F$ [1,9] = 11.573, $p = 0.008$, partial $\eta^2 = 0.563$) and SF ($F$ [2.2,20.3] = 138.156, $p < 0.001$, partial $\eta^2 = 0.939$) and a significant interaction between eye and condition ($F$ [1,9] = 15.228, $p = 0.004$, partial $\eta^2 = 0.629$). Further Bonferroni-corrected pairwise comparisons showed no significant difference between balanced CSF and linear summation in NDE ($p = 0.05$) nor DE ($p = 0.476$).

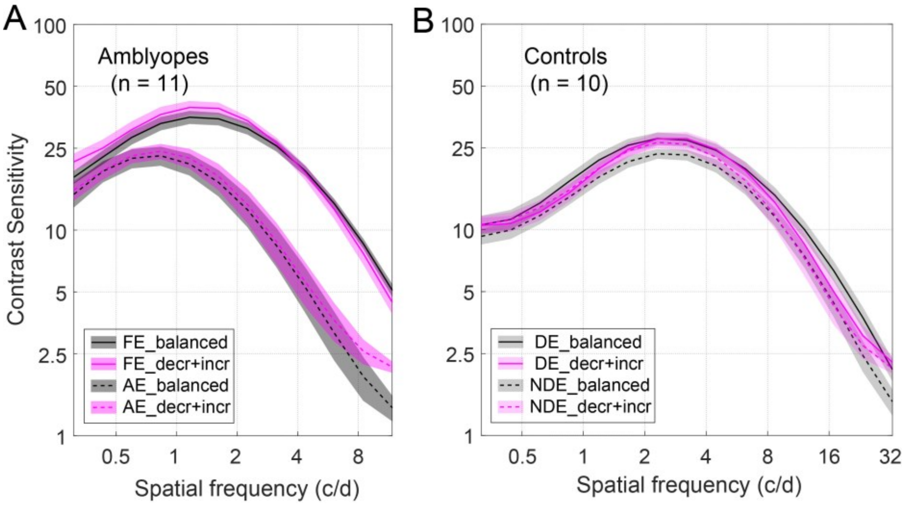


**Fig. S4.** Balanced CSF (gray) and linearly summation from increment and decrement CSF (pink) of amblyopes (left) and controls (right). The solid lines represent FE and NDE, the dotted lines represent AE and DE. The shaded regions represent ± SE.
